# Health-Related Social Needs Facing Youth With Nonalcoholic Fatty Liver Disease

**DOI:** 10.1097/PG9.0000000000000153

**Published:** 2021-12-10

**Authors:** Sarah Orkin, Toshifumi Yodoshi, Qin Sun, Lin Fei, Syeda Meryum, Sanita Ley, Ana Catalina Arce-Clachar, Kristin Bramlage, Stavra Xanthakos, Robert Kahn, Andrew F. Beck, Marialena Mouzaki

**Affiliations:** From the * Division of Gastroenterology, Hepatology and Nutrition, Cincinnati Children’s Hospital Medical Center, Cincinnati, OH; † Department of Pediatrics, University of Cincinnati College of Medicine, Cincinnati, OH; ‡ Division of Biostatistics and Epidemiology, Cincinnati Children’s Hospital Medical Center, Cincinnati, OH; § Division of Behavioral Medicine and Clinical Psychology, Cincinnati Children’s Hospital Medical Center, Cincinnati, OH; ∥ Division of General and Community Pediatrics, Cincinnati Children’s Hospital Medical Center, Cincinnati, OH; ¶ James M. Anderson Center for Health Systems Excellence, Cincinnati Children’s Hospital Medical Center, Cincinnati, OH; # Division of Hospital Medicine, Cincinnati Children’s Hospital Medical Center, Cincinnati, OH.

**Keywords:** fatty liver, food insecurity, socioeconomic status

## Abstract

The objective of this study was to identify the prevalence of health-related social needs among youth with nonalcoholic fatty liver disease.

**Methods:**

Retrospective review of prospectively administered health-related social needs questionnaires from Steatohepatitis Clinics.

**Results:**

Patients with nonalcoholic fatty liver disease (n = 271) were predominantly male (72%) and non-Hispanic (68%). The most common unmet need was food insecurity (13%, n = 36). Families who endorsed food insecurity at the first visit were 27-fold more likely to have unmet health-related social needs persist at subsequent visits than those who were food secure at their first visit (95% confidence interval, 6.7-111).

**Conclusions:**

Screening for social, economic, and environmental needs may identify previously unrecognized family challenges and may enhance intervention delivery, inform resource allocation, and improve outcomes.

## Abstract 2

Supplemental Digital Content is available in the text.

## INTRODUCTION

Nonalcoholic fatty liver disease (NAFLD) poses a significant burden on the health and quality of life of children globally (1). The treatment for children remains lifestyle modification targeted towards achieving a healthier weight status (2,3). It is estimated that nonadherence to prescribed regimens may affect more than half of children with chronic gastrointestinal conditions such as inflammatory bowel disease and celiac disease (4,5). The efficacy of lifestyle counseling provided in the healthcare setting may be enhanced by understanding the health-related social needs faced by families related to parental health literacy, family food security status, housing instability, and neighborhood safety.

Social determinants of health are defined by the World Health Organization as “the conditions in which people are born, grow, work, live, and age, and the wider set of forces and systems shaping the conditions of daily life (6).” The relationships between the social determinants of health and NAFLD risk factors have been previously investigated, and a recent study demonstrated that cumulative exposure to socioeconomic disadvantage in childhood was associated with the risk of developing NAFLD in adulthood (7–11). Orkin et al (12) reported an association between neighborhood-level socioeconomic deprivation with earlier onset of pediatric NAFLD. However, the prevalence and type of specific health-related social needs faced by children with NAFLD remain unclear. Elucidating these patient-level health-related social needs is critical as they may pose challenges to implementing the recommended lifestyle interventions critical to NAFLD regression.

The objective of this study was to describe the prevalence and distribution of health-related social needs faced by youth with NAFLD.

## METHODS

### Study Design

Retrospective review using convenience sampling of prospectively collected data from the Steatohepatitis Center of Cincinnati Children’s Hospital Medical Center after Institutional Review Board approval.

### Cohort

Patients included were seen at the Steatohepatitis Center for presumed or histologically confirmed NAFLD, whose caregivers had been administered at minimum one social needs questionnaire between September 1, 2018, and October 10, 2019, and who were 4 to 21 years old at time of initial questionnaire completion (Supplemental Figure 1, http://links.lww.com/PG9/A64). Presumed NAFLD was defined as persistently elevated serum alanine aminotransferase (ALT) levels (>50 U/L for >3 months) and/or imaging consistent with hepatic steatosis (hepatic fat fraction ≥5% on magnetic resonance imaging proton density fat fraction) in the absence of other causes of liver disease.

### Exclusion Criteria

Patients were excluded if they were liver transplant recipients or had had preceding weight loss surgery. Given that this was a questionnaire used to augment patient care in the clinical setting, completion was not required, and if a patient/family opted to leave it blank, they were not directed to complete it.

### Health-Related Social Needs Screening

The “Events and Stressors Questionnaire,” a 17-question tool related to social, economic, and environmental needs, was given to all caregivers /patients (if ≥ 18 years old) upon arrival to the Steatohepatitis Clinic (Supplemental Figure 2, http://links.lww.com/PG9/A64), as part of routine clinical practice. The questionnaire was first used in the NAFLD clinic as of September 2018. Categorical questions addressed health-related social needs including food insecurity, access to fresh produce, housing difficulties, travel difficulties, neighborhood safety, medication affordability, and parental education. Some participants manually entered information about educational attainment (ie, if elementary school was highest level completed), and this was included in the results. The first 2 questions included in the questionnaire comprised an independently validated screening tool for food insecurity (13). The questionnaires were available in both English and Spanish. If a caregiver /patient endorsed difficulty reading or understanding, they were assisted by either clinical staff or family members, if desired. If a family reported the presence of an actionable health-related social need, the social work team was notified for assistance.

### Repeat Questionnaires

The number and distribution of health-related social needs from each repeat questionnaire obtained at follow-up clinic visits were assessed. Patients were then divided into 3 groups for analysis based on the change in the number of needs identified over time: those who persistently reported lack of health-related social needs, those who persistently had “any,” and those who fluctuated between having no health-related social needs and having “any” present.

### Clinical Data

Clinical characteristics including demographic (race, ethnicity, sex) and anthropometric values were obtained from the electronic health record within 30 days of questionnaire completion. Laboratory values within 30 days of clinic visit were recorded, including: ALT (U/L), aspartate aminotransferase (U/L), gamma-glutamyltransferase (U/L), alkaline phosphatase (U/L), and glycated hemoglobin A1c (%).

### Statistical Analysis

Descriptive statistics were employed to characterize the distribution of key clinical, demographic, and social needs variables, using means with SDs or medians with ranges for continuous variables and proportions for categorical variables. When comparing groups, Student *t* test, chi-square test (or Ridit analysis in cases of 3 or more ordered response categories), Wilcoxon rank-sum testing, or Fisher exact test were used. For analysis of repeat questionnaires, logistic regression with proportional odds modeling was used to explore associations between status of various health-related social needs (as univariate predictors) and persistence of health-related social needs. As outlined above, 3 different states were defined in a proportional odds model: those who persistently reported no health-related social needs, those who persistently had “any,” and those who fluctuated between the 2 states. Odds ratio was defined for each individual health-related social need as the ratio of odds when reporting the presence versus odds when reporting the absence of each health-related social need. An exploratory pairwise correlation coefficient analysis was used to determine the correlation between each health-related social need as a predictor of the presence of another health-related social need. SAS 9.4 (Cary, NC) was used for analysis.

## RESULTS

### Patient Characteristics

**FIGURE 1.**
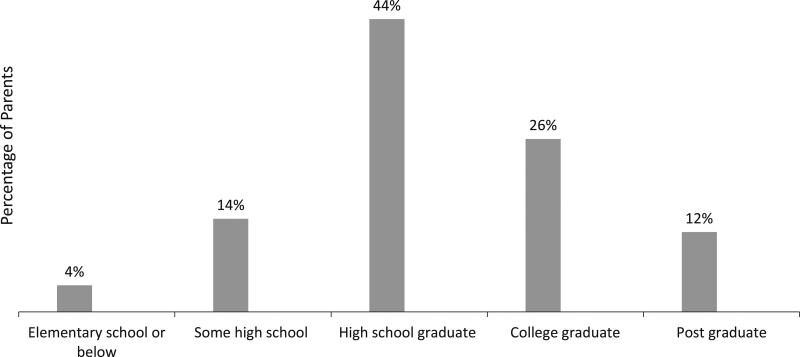
Maximum educational attainment of parents of patients (n = 250).

From the Steatohepatitis Clinic, 271 patients’ first-time responses to the questionnaire were collected. The majority were male (72%) and non-Hispanic (68%), and mean age was 13 years (Table 1). Among the participants who provided information regarding parental education (n = 250), the majority (82%, n = 205) had a high school degree or greater, while 18% (n = 45) had not graduated high school (Fig. 1).

**TABLE 1.** Baseline characteristics of first time questionnaire respondents

Variable	NAFLD patients, N = 271
Demographics	
Male sex, n (%)	196 (72)
Age, y, mean (SD)	13 (3.5)
English as primary language, n (%)	202 (75)
Hispanic ethnicity, n (%)	87 (32)
Race, n (%)	
White	190 (70)
Black	16 (6)
Other/not reported	65 (24)
Maternal age at birth, y, mean (SD)	28 (6.6)
Parental status—married, n (%)*	134 (55)
Parents without high school degree, n (%)†	
Total	45 (18)
Hispanic	31 (12)
Non-Hispanic	14 (6)
Parents with graduate school degree†	29 (12)
Clinical/laboratory	
BMI *z* score, mean (SD)	2.37 (0.4)
ALT (U/L), median (IQR)	59 (10–802)
AST (U/L), median (IQR)	34 (6–296)
GGT (U/L), median (IQR)	31 (3–299)
Alk Phos (U/L), median (IQR)	177 (42–469)
Social needs, n (%)	
Food insecurity	36 (13)
Lack safe outdoor space	19 (7)
Any needs present	89 (33)

Alk Phos = alkaline phosphatase; ALT = alanine aminotransferase; AST = aspartate aminotransferase; BMI = body mass index; GGT = gamma-glutamyltransferase; IQR = interquartile range; NAFLD = nonalcoholic fatty liver disease.

*243 respondents.

†250 respondents.

**FIGURE 2.**
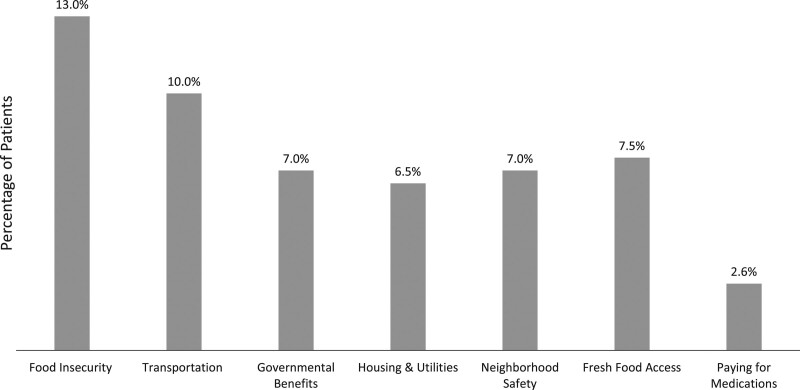
Breakdown of types of health-related social needs present.

One-third of the 271 respondents (n = 89) reported the presence of at least 1 social need, and 14% of patients (n = 39) had 2 or more needs present. The most commonly reported unmet need in the NAFLD cohort was food insecurity (13%, n = 36), followed by transportation difficulties (10%, n = 28) (Fig. 2).

When the cohort was dichotomized into those with no health-related social needs and those with any such needs, patients experiencing “any” need were younger (13 versus 14 years old; *P* = 0.008), less likely to be English speaking (64% versus 83%; *P* = 0.001), and less likely to have parents with a college degree or higher (17% versus 30%; *P* = 0.028; Supplemental Table 1, http://links.lww.com/PG9/A64). There was no significant difference between presence or absence of need and biochemical evidence of liver disease severity (Supplemental Table 1, http://links.lww.com/PG9/A64).

When the cohort (n = 271) was stratified by ethnicity (Hispanic versus non-Hispanic), patients of Hispanic ethnicity (n = 87) were more likely to be younger (12 versus 15 years; *P* < 0.001) and to have a higher ALT (98 versus 73 U/L; *P* = 0.040) despite the same degree of obesity as the non-Hispanic patients (body mass index *z* score 2.27 versus 2.43; *P* = 0.006). Parents of Hispanic ethnicity were more likely to lack a car (12% versus 3.5%; *P* = 0.010), to lack a high school degree (41.9% versus 8.3%; *P* < 0.001), and to endorse utility problems (11.5% versus 4.6%; *P* = 0.042). The remaining demographic, educational, and laboratory differences are shown in Supplemental Table 2 (http://links.lww.com/PG9/A64). Despite these differences, there was no significant difference in the number of health-related social needs per patient or the prevalence of those needs between Hispanic and non-Hispanic patients.

### Correlation Between Individual Health-Related Social Needs

Reported food insecurity was correlated with the presence of multiple other health-related social needs, including transportation distance, governmental benefits, and utilities (Supplemental Table 3, http://links.lww.com/PG9/A64).

### Change in Unmet Health-Related Social Needs Over Time

Of the 271 patients with NAFLD completing a baseline questionnaire, 99 patients completed repeat questionnaires (at least once) at subsequent visits. These 99 patients generated 229 questionnaires across multiple follow-up visits (Supplemental Figure 1, http://links.lww.com/PG9/A64). Of these 99 patients, 34% (n = 34) had 1 or more health-related social need at baseline. Across repeated assessments, half (n = 49) remained without any unmet needs, 18% (n = 18) had persistence of health-related social needs time, and 32% (n = 32) fluctuated between absence and presence of health-related social needs. Of the patients reporting fluctuation in health-related social needs status, all but 1 (97%, n = 31) reported the absence of any health-related social needs on at least 1 occasion.

Certain health-related social needs were more likely to persist across repeated evaluations. These included food insecurity, governmental benefits issues, and issues with medication affordability (Table 2). Families who endorsed food insecurity at the first visit were 27-fold more likely to have unmet health-related social needs persist at subsequent visits than those who were food secure at their first visit (95% confidence interval [CI], 6.7-111). The odds of health-related social needs persisting over repeated evaluations were 20-fold higher in those with trouble paying for medications as compared to those without trouble (95% CI, 1.7-250), 10-fold higher in those with Social Security Income concerns as compared to those without Social Security Income problems (95% CI, 2.2-50), and 7-fold higher in those with Special Supplemental Nutrition Program for Women, Infants, and Children or Supplemental Nutrition Assistance Program concerns than in those without (95% CI, 1.4-35.7).

**TABLE 2.** Health-related social needs associated with increased likelihood of future social need persistence across repeated evaluations

Health-related social need present	Odds ratio (any vs none) (95% confidence limits)	*P*
Food insecurity	0.037 (0.009-0.149)	<0.001
Trouble paying for medications	0.050 (0.004-0.606)	0.019
SSI concerns	0.097 (0.020-0.460)	0.003
Lacking car	0.135 (0.015-1.256)	0.079
WIC or SNAP concerns	0.141 (0.028-0.715)	0.018
Travel distance to clinic	0.174 (0.025-1.197)	0.076
Housing and utility problems	0.212 (0.038-1.189)	0.078
Safe space to play outdoors	4.859 (0.986-23.954)	0.052
Number of weekly meals consumed at home	1.009 (0.806-1.263)	0.941

SNAP = Supplemental Nutrition Assistance Program; SSI = Social Security Income; WIC = Special Supplemental Nutrition Program for Women, Infants, and Children.

## DISCUSSION

Our findings demonstrate a substantial prevalence of patient-reported unmet health-related social needs among children presenting for evaluation in NAFLD. As it pertains to NAFLD, these reported social needs may directly affect the success of lifestyle counseling, including food insecurity (13%) and lack of safe outdoor space for playing or exercising (7%). In addition, the parents of 19% of patients reported not having graduated high school, suggesting lower health literacy as another possible barrier to the success of the counseling received in clinic. In those with repeated assessments, half have evidence of either persistent or intermittent exposure to socioeconomic barriers. Certain barriers, such as food insecurity, are more likely to persist over time.

The North American Clinical Practice Guidelines for the Diagnosis and Treatment of NAFLD in Children recommends that lifestyle intervention counseling is offered to all overweight or obese children with NAFLD (2). However, the presence of many of the aforementioned health-related social needs may impair the ability of a family to follow the recommendations made in clinic, as they require access to safe outdoor space for physical activity, nutrient-rich food availability, and sufficient health literacy. Some of the socioeconomic barriers present may be modifiable (ie, assisting with transportation difficulties), while others may not (ie, lack of high school education) and may require modification of healthcare delivery (ie, enhanced communication). Knowledge of these barriers allows medical recommendations to be tailored; for example, when access to outdoor space for physical activity is limited, the clinician can recommend exercises that can be done indoors. Similarly, when the cost of fresh fruit and vegetables is prohibitive to a family, the discussion can be focused more on portion control and elimination of sugar-sweetened beverages (14). This approach may optimize the success of recommended lifestyle interventions.

Food insecurity was the most common health-related social need encountered, seen in 13% of our patients. Food insecurity, defined by Feeding America as “a lack of consistent access to enough food for every person in a household to lead an active, healthy life” correlated with the presence of multiple other health-related social needs (15). It is crucial to determine the food security status of patients with NAFLD given new insights into the role of food insecurity in NAFLD. A recent study by Golovaty et al (16) examined 2627 adults and found that those with food insecurity were more likely to have NAFLD, while Tamargo et al (17) reported that in adults, food insecurity was associated with more advanced fibrosis and attenuated the risk of NAFLD in those with obesity. These findings, in conjunction with our recent geospatial work outlining that children from more deprived neighborhoods may be at risk for earlier onset of NAFLD, suggest that pathophysiologic mechanisms may link food insecurity and liver disease severity, such as alteration in cortisol levels and hunger cues resulting from food insecurity that impacts insulin sensitivity and lipolysis from the peripheral tissue (12). Furthermore, in the presence of food insecurity, one’s diet may include more proinflammatory macronutrients such as fructose and saturated fat. The emerging association in adults between food insecurity and NAFLD severity provide rationale for ongoing research in this field.

Through analysis of repeated screening for health-related social needs over time, we found that 32% of patients fluctuated between the presence and absence of health-related social needs on repeat questionnaire administration and that most of these patients reported no health-related social needs present on at least 1 occasion. As such, 1-time screening of patients does not effectively capture a realistic view of the home environment and should be repeated serially. Importantly, the presence of several health-related social needs at the time of first assessment were associated with an increased likelihood that health-related social needs would persist over time on subsequent assessments. Therefore, knowledge of these social needs at the time of first clinic visit should alert providers that these patients are at risk for ongoing health-related social needs and may warrant more robust follow-up and/or resource allocation.

Given that food insecurity was both the most prevalent unmet need identified and also one of the unmet needs associated with socioeconomic risk persistence over time, it is reasonable to screen all families presenting to clinic for the evaluation of NAFLD for food insecurity. This practice would be in line with guidelines from the American Academy of Pediatrics, which recommends universal screening for food insecurity using the 2-question tool developed by Hager et al (13) followed by referral to the appropriate community resources (18).

Our study has several limitations. Completion of questionnaires was voluntary, which may have introduced a selection bias. Furthermore, while responses indicating the presence of health-related social needs were reviewed by the medical staff in clinic with the family for confirmation, screens that did not report the presence of a health-related social need were not reviewed with the family, potentially biasing the results toward the null.

Screening all patients with NAFLD for the presence of health-related social needs will allow contextual knowledge that can aid in targeted interventions, resource allocation and may increase efficacy of both lifestyle modification counseling by providers and implementation by families. Our findings highlight the need for further research to understand the manner and frequency with which to screen patients with NAFLD for health-related social needs and the mechanisms through which such unmet needs affect disease outcome.

## Supplementary Material


