# Supplementary material for: Health-Related Social Needs Facing Youth With Nonalcoholic Fatty Liver Disease
Source: JPGN Rep. 2021 Dec 10;3(1):e153. doi: 10.1097/PG9.0000000000000153 (PMC9191845; doi:10.1097/PG9.0000000000000153)
Supplement: Supplementary file 1 [file pg9-3-e153-s001.pdf]

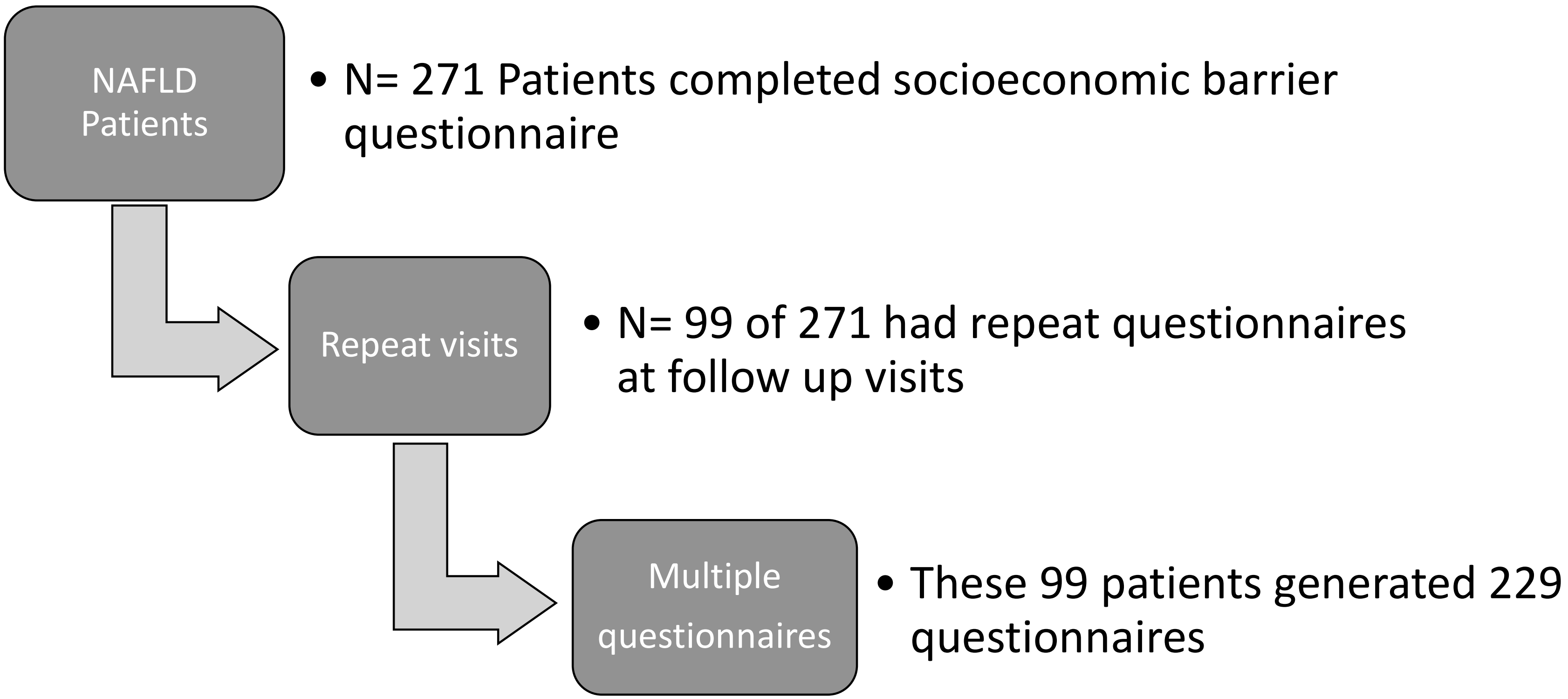

Supplemental Figure 1. Breakdown of Respondents

Cincinnati Children's
changing the outcome together

Steatohepatitis Clinic
Events and Stressors Questionnaire

Name: ____________________
DOB: ____________________
MRN: ____________________

To be completed by Parent/Caregiver.

Date: ____________________

Name of person completing form: ____________________ Relationship to patient: ____________

Every family faces different stressors. We ask all families about stressors because we may be able to help.

We will keep this information confidential.

1. Within the past 12 months, did you/your family worry whether your food would run out before you got money or SNAP/food stamps to buy more? ☐ Yes ☐ No
2. Within the past 12 months, did the food you/your family bought not last and you didn't have money to get more? ☐ Yes ☐ No
3. Can you afford to buy fresh fruit and vegetables? ☐ Yes ☐ No
4. Are you currently having any problems with:
   - WIC, SNAP/food stamps ____________________ ☐ Yes ☐ No
   - Medical card/Insurance, SSI ____________________ ☐ Yes ☐ No
   - Utilities ____________________ ☐ Yes ☐ No
5. How many people live in your home? __________
6. Are you currently being threatened with eviction or losing your home? ☐ Yes ☐ No
7. Since your last appointment, have you had trouble paying for medications or have you chosen not to fill a medication due to cost? ☐ Yes ☐ No
8. Do you currently have trouble getting to doctor's appointments or to the pharmacy due to:
   - Distance to travel ____________________ ☐ Yes ☐ No
   - Don't have a car ____________________ ☐ Yes ☐ No
   - Other? ____________________
9. Are you able to get to a safe place for your children to play outdoors if you wanted to? ☐ Yes ☐ No
10. Where does your family most often purchase food from?
    ☐ Grocery store ☐ Corner store ☐ Other: ____________________
11. Are you able to get to a grocery store easily? ☐ Yes ☐ No
12. During the school year, how many meals per day does the school provide to your child? __________
13. How many nights per week (on average) does your family eat a meal prepared at home? __________
14. How old were both parents when child was born? Mother: __________ Father: __________
15. Parental marital status? ☐ Single ☐ Married ☐ Divorced
16. Does anyone in the home smoke? ☐ Yes ☐ No
17. What is the highest level of education for either parent of the patient?
    ☐ Some high school ☐ Completed high school
    ☐ Some college ☐ Completed college ☐ Post graduate

DO NOT WRITE BELOW LINE, *office use only:*

Form reviewed by Signature/Credentials Printed Name Date Time

Supplemental Table 1. Comparison of patient characteristics between cohorts with and without health-related social needs present

Variable	Any Need Present (n=89)	No Need Present (n=182)	P -value
**Patient Age, mean years (SD)**	14 (3.7)	13 (2.9)	0.008
**Maternal Age, mean years (SD)**	28 (6.5)	28 (6.7)	0.817
**English Language, n (%)**	55 (64%)	147 (83%)	0.001
**Male Sex, n (%)**	58 (67%)	130 (74%)	0.312
**Hispanic Ethnicity, n (%)**	34 (40%)	53 (30%)	0.246
**No High School degree, n (%)**	19 (25%)	26 (15%)	0.067
**High School Degree, n (%)**	38 (50%)	72 (42%)	0.256
**College Degree, n (%)**	13 (17%)	52 (30%)	0.028
**Graduate School Degree, n (%)**	7 (9%)	22 (13%)	0.409
**ALT (U/L), mean (SD)**	84 (78)	80 (93)	0.770
**AST (U/L), mean (SD)**	50 (45)	45 (42)	0.388
**GGT (U/L), mean (SD)**	48 (37)	43 (42)	0.512
**Alk Phos (U/L), mean (SD)**	218 (114)	191 (107)	0.076
**BMI (kg/m^2), mean (SD)**	34.5 (7.7)	34.2 (7.1)	0.740

ALT – Alanine Aminotransferase, Alk Phos – Alkaline Phosphatase, AST – Aspartate Aminotransferase, BMI- Body Mass Index, GGT – Gamma-Glutamyltransferase,

BMI- Body Mass Index, ALT – Alanine Aminotransferase, AST – Aspartate Aminotransferase, GGT – Gamma-Glutamyltransferase, Alk Phos – Alkaline Phosphatase

Supplemental Table 2. Comparison of baseline characteristics between Hispanic and Non-Hispanic patients

	Variable	Hispanic Ethnicity N=87	Non-Hispanic Ethnicity N=175¥	P -value
Demographic	Age at visit; mean years, (SD)	12 (2.9)	15 (3.4)	< 0.001
	Parents Lacking High School Degree*	41.9%	8.3%	<0.001
	One parent with High School Degree	40.5%	44.6%	0.553
	One parent with College Degree	14.9%	31.0%	0.009
	One parent with Graduate Degree	2.7%	16.1%	0.003
	Maternal Age at patient's birth, mean years, (SD)	27 (6.7)	29 (6.5)	0.038
	Paternal Age at patient's birth, mean years, (SD)	29 (7.8)	31 (7.0)	0.021
Clinical	BMI z-score	2.27 (0.36)	2.43 (0.47)	0.006
	AST (U/L); mean years, (SD)	52 (51)	44 (38)	0.179
	ALT (U/L); mean years, (SD)	98 (124)	73 (62)	0.040
	GGT (U/L); mean years, (SD)	43 (34)	45 (45)	0.699
	Alk Phos (U/L); mean years, (SD)	255 (108)	173 (101)	<0.001
Social Needs	Any Social Need present	39.1%	29.7%	0.128
	Number of barriers; mean, (SD)	0.74 (1.2)	0.59 (1.2)	0.367
	Food Insecurity	11.9%	12.6%	0.866
	SSI Problems	7.3%	4.0%	0.258
	Utility Problems	11.5%	4.6%	0.042
	Lacking car	12%	3.5%	0.010
	WIC or SNAP problems	4.7%	3.4%	0.615
	Trouble paying for meds	2.4%	2.9%	0.831
	Safe outdoor space	92.7%	93.0%	0.921

¥ Not all patients provided an ethnicity, thus the total N ≠ 271

* 74 Hispanic and 168 non-Hispanic answered, respectively

ALT – Alanine Aminotransferase, Alk Phos – Alkaline Phosphatase, AST – Aspartate Aminotransferase, BMI- Body Mass Index, GGT – Gamma-Glutamyltransferase, SNAP – Supplemental Nutrition Assistance Program, SSI – Social Security Income, WIC – Special Supplemental Nutrition Program for Women, Infants and Children,

Supplemental Table 3. Association Between Health-Related Social Needs in 271 Patients with NAFLD

Variables	Predictors	Correlation Coefficient	P-value
Food Insecurity	Utility problems	0.37	< 0.001
	SSI problems	0.28	< 0.001
	Travel distance to clinic	0.26	< 0.001
	WIC or SNAP problems	0.24	0.002
	Lack of car	0.07	0.225
	Smoker in the home	0.07	1.00
	Neighborhood safety	0.01	1.00
	Paying for medications	0.01	1.00
WIC or SNAP problems	Utility problems	0.29	0.001
	Travel distance to clinic	0.25	0.007
	Lack of car	- 0.05	1.00
	Neighborhood safety	- 0.028	0.499
SSI problems	Utility problems	0.31	< 0.001
	Travel distance to clinic	0.03	0.466
	Lack of car	0.03	0.464
	Neighborhood safety	-0.01	0.499
Utility problems	Travel distance to clinic	0.31	< 0.001
	Lack of car	0.17	0.008
Processed food consumption	Neighborhood safety	0.25	< 0.001
	Utility problems	0.13	0.018
	Food Insecurity	0.09	0.069
	Travel distance to clinic	0.05	0.319
	WIC or SNAP problems	0.05	0.206
Travel distance to clinic	Neighborhood safety	- 0.01	1.00
	Paying for medications	0.07	0.305
Lack of car	Neighborhood safety	- 0.06	0.293
	Paying for medications	- 0.04	1.00

SNAP – Supplemental Nutrition Assistance Program, SSI – Social Security Income, WIC – Special Supplemental Nutrition Program for Women, Infants and Children
